# Comparison of eosin and fluorescein conjugates for the photoinitiation of cell-compatible polymer coatings

**DOI:** 10.1371/journal.pone.0190880

**Published:** 2018-01-08

**Authors:** Jacob L. Lilly, Anuhya Gottipati, Calvin F. Cahall, Mohamed Agoub, Brad J. Berron

**Affiliations:** Department of Chemical and Materials Engineering, University of Kentucky, Lexington, Kentucky, United States of America; Brandeis University, UNITED STATES

## Abstract

Targeted photopolymerization is the basis for multiple diagnostic and cell encapsulation technologies. While eosin is used in conjunction with tertiary amines as a water-soluble photoinitiation system, eosin is not widely sold as a conjugate with antibodies and other targeting biomolecules. Here we evaluate the utility of fluorescein-labeled bioconjugates to photopolymerize targeted coatings on live cells. We show that although fluorescein conjugates absorb approximately 50% less light energy than eosin in matched photopolymerization experiments using a 530 nm LED lamp, appreciable polymer thicknesses can still be formed in cell compatible environments with fluorescein photosensitization. At low photoinitiator density, eosin allows more sensitive initiation of gelation. However at higher functionalization densities, the thickness of fluorescein polymer films begins to rival that of eosin. Commercial fluorescein-conjugated antibodies are also capable of generating conformal, protective coatings on mammalian cells with similar viability and encapsulation efficiency as eosin systems.

## Introduction

The eosin Y photopolymerization system has been widely employed for radical polymerization in aqueous biological environments using low intensity, visible light irradiation [1–5]. Eosin is a xanthene dye photosensitizer which is excitable by visible light, and initiates polymerization when paired with a triethanol amine (TEA) coinitiator [6, 7]. Upon irradiation, the eosin is excited to the triplet state and abstracts hydrogen from TEA to yield a protonated eosin radical a protonated TEA radical. The TEA radical then initiates polymerization, while the eosin radical is regenerated through disproportionation with an inhibiting radical species [3, 8]. The cyclic regeneration of eosin enables the formation of polymer in systems where inhibiting species are ~1000 fold more concentrated than eosin [8]. Importantly, this system has been used to initiate the bulk gelation of cell laden tissue engineering scaffolds with reported high cell viabilities [9]. Eosin Y has also been functionalized to surfaces to form hydrogel sites of protein recognition on microarray[10–12] and cellular substrates [1], and has shown remarkable efficiency and sensitivity in response to ultralow analyte densities [10, 11, 13–15].

Our group has developed eosin Y for the photopolymerization of hydrogel film coatings on living cell membrane substrates for applications in rare cell sorting[16] and immunoisolation of transplantable cells [17]. This strategy is unique from previous micron-scale coating techniques where mammalian cells are soaked in an aqueous eosin solution [2, 18]. Here, our strategy is based on labeling cell surface proteins with eosin for hydrogel polymerization at the cell membrane. Antibodies are coupled with eosin labels to localize eosin to the cell membrane of antigen positive cells. When these eosin-labeled cells are irradiated in a monomer and triethanol amine solution, polymerization proceeds only at the eosin-primed cell surfaces, yielding a ~100 nm polymer coating at high cell viabilities (~90%) [16, 19, 20]. A critical limitation to this coating approach is the requirement of each researcher to prepare custom eosin-biomolecule conjugates to localize the initiator at the cell membrane.

Fluorescein is another xanthene dye which is capable of radical generation in aqueous media and polymerization [3, 5, 8]. Fluorescein functions in a similar manner as eosin, where photopolymerization is initiated through hydrogen abstraction in the triplet state. The specifics of eosin polymerization are well described in the literature [2, 5, 6, 8, 18, 21–24] while reports of fluorescein polymerization are largely comparative to eosin [8, 25]. Fluorescein has also exhibits cyclic dye regeneration, but has a lesser capacity to overcome inhibiting species [8, 25]. Polymerization of a hydrogel was slower using fluorescein than an equivalent eosin initiation system, and surface tethered fluorescein conjugates have shown that fluorescein is less effective in reaching gelation at low surface densities when compared to eosin [8, 25]. While the commercial availability of eosin-isothiocyanate (EITC) labeled antibodies is extremely limited, fluorescein-isothiocyanate (FITC) labeling is one of the most common label formats and is commercially available for many antibodies.

In this study, we quantitatively compare the potency of these xanthene photosensitizers to enact surface-mediated polymer film formation on cells. While eosin and fluorescein have previously been investigated simultaneously for polymerization kinetics in aqueous media [5, 8, 25], no prior study evaluates commercially-available FITC-conjugated antibodies or the use of fluorescein in the pH neutral, ultra-low TEA concentrations required for cell viability. Our study evaluates film thickness generated by fluorescein or eosin under conditions conducive to live cell encapsulation using photoinitiator-functionalized planar glass microarrays. These microarrays allow for excellent control over reaction condition and high reproducibility, which is not afforded by direct experimentation on cultured cell populations. For live cell studies, we target EGFR receptors on A549 cells and evaluate the viability of cells after surface-mediated coating formation by FITC or EITC initiation. We also challenge the coated cells with deionized water to determine which cells are sufficiently reinforced to prevent expansion and lysis from hypotonic expansion. Our results show that FITC-antibodies are capable of initiating cell coatings and demonstrate an exciting path forward in translating our technology to a broad range of applications. Further, for situations in which custom protein labeling is necessary, FITC is approximately 50-fold cheaper by mass than EITC, and is often already stocked in the labs of many researchers in biology-related fields, potentially allowing for easier integration of photopolymer coating technologies.

## Materials and methods

### Materials

Bovine serum albumin (BSA), biotinylated bovine serum albumin (bio-BSA), streptavidin, streptavidin-Cy3 conjugate (SA-Cy3), triethanolamine (TEA), Zeba 7K Da molecular weight cutoff desalting spin columns, and Fluosphere fluorescent nanoparticles (20 nanometer diameter, carboxylate-terminated, nile red fluorescence) were purchased from Thermo Fisher Scientific. Phosphate buffered saline (PBS), fluorescein isothiocyanate isomer I, eosin-5-isothiocyanate (EITC), Cy3 NHS ester, poly(ethylene glycol) diacrylate (M_n_~575 Da), monomethyl ether hydroquinone dehibiting columns, vinyl-2-pyrrolidinone, thiazoyl blue tetrazolium bromide (MTT), and dimethyl sulfoxide (DMSO) were purchased from Sigma Aldrich. Epoxy-functionalized, glass microarray slides were supplied by Array-It Corp. A non-small cell lunger cancer line A549 was purchased from ATCC. RPMI-1640 growth media, penicillin/streptomycin, and trypsin/EDTA (0.25% solution) were purchased from Gibco. Advantage grade fetal bovine serum (FBS) was purchased from Atlanta Biologicals. The primary antibody, anti-human EGFR was purchased from Biolegend. The secondary antibody, FITC-labeled horse anti-mouse IgG was purchased from Vector Labs.

### Protein microarray fabrication

Epoxy slides were first washed with ethanol, dried with pressurized N_2_, and aligned on the platform of an Affymetrix 417 arrayer. Solutions of bio-BSA were prepared in phosphate-buffered saline (PBS 1X) at 12 serially diluted concentrations (1000, 400, 160, 64, 25.6, 10.2, 4.1, 1.64, 0.66, 0.26, 0.1, and 0 μg/mL) with the balance consisting of unconjugated BSA to keep the total protein concentration at 1000 μg/mL for all solutions. These solutions were transferred to a 96 well plate and loaded into the arrayer. While maintaining the arrayer at 60% humidity, two identical sets of 24 spot arrays printed on each slide, and each array contained two spots of each bio-BSA concentration. The array locations were set for each slide to be centered within the wells of Whatman Chip Clip Fast-Slides. All printed microarrays were then allowed to dry overnight and stored at room temperature in the dark for later experiments.

### Streptavidin-photoinitiator synthesis

Two xanthene dye photoinitiators (EITC and FITC) were conjugated to streptavidin similarly to a previously published protocol[10] at identical molar ratios, with the minor change of carrying out the reaction in a 0.1 M carbonate/bicarbonate buffer at pH~10. Protein conjugates were purified in PBS 1X using a Zeba desalting spin column.

### UV/vis spectroscopy of fluorescent dye conjugates

The degree of labeling of fluorescent dye-streptavidin conjugates used in this study (SA-EITC, SA-FITC, and SA-Cy3) was determined by UV-Vis spectrophotometry. Lyophilized streptavidin, EITC, FITC, and Cy3 NHS ester were each separately dissolved in PBS at serial dilutions, and absorbance values were measured using a NanoDrop 2000 (Thermo Scientific) UV-Vis spectrophotometer. Absorbance values were obtained at wavelengths corresponding to peak absorption for each molecule as follows: streptavidin– 280 nm, EITC– 530 nm, FITC– 495 nm, Cy3–550 nm. Data at each concentration were obtained in triplicate, and standard curves were generated from absorbance values vs. concentration by linear regression. The UV-Vis absorbance spectra of fluorescent dye conjugates dissolved in PBS were also obtained in triplicate. Using the generated linear fit equations detailed in our previous study [26], the degree of labeling for each fluorescent conjugate was then determined to be 2.2 (SA-Cy3), 5.1 (SA-EITC), and 4.3 (SA-FITC). Additionally, absorbance peaks at 280 nm were used to determine the overall protein concentration in stock dye solutions, and solutions were adjusted to between 0.5 and 1 mg/ml for storage of all dye conjugates.

### Specific tagging and photopolymerization of initiator-protein arrays

Biotinylated-BSA printed slides were loaded into Whatman Fast-Slide microarray incubation chambers. Each array well was first quickly rinsed once with PBS containing 1 mg/ml BSA (PBSA) to remove any unbound bio-BSA remaining from the printing process, then incubated with PBSA for 40 minutes to block unreacted epoxy groups. All incubations were done at room temperature protected from light. Unless otherwise noted, each rinsing and incubation solution was contacted with the array slide at a volume of 500 μL. After removing the blocking solution, a streptavidin-initiator (SA-EITC or SA-FITC) solution was prepared at 25 μg/ml in PBSA and contacted with each array well for 30 minutes. This solution was then removed and the slides were washed once with PBSA for 3 minutes, and then twice more with PBS for 3 minutes each. A cell compatible monomer precursor is prepared fresh for each experiment containing 420 mM PEG diacrylate-575, 21 mM TEA and 35 mM VP in PBS and adjusted to a pH of ~7.5. The unconstrained formulation consisted of 420 mM PEGDA-575, 210 mM TEA and 35 mM VP in deionized water. All solutions were purged with ultra-pure N_2_ by gentle bubbling through a syringe needle for 10 minutes. 325 μL of monomer precursor solution is then immediately pipetted into the array well, and placed into a purging chamber constructed out of a modified polystyrene petri dish, and the entire apparatus is further purged for 5 minutes. Arrays were then irradiated with a collimated LED lamp (M530L3, Thorlabs) with maximum intensity at 530 nm for 10 minutes at either 20 mW/cm^2^ or 30 mW/cm^2^. After irradiation, each slide was rinsed 5 times with deionized water and stored in a slidebox to dry overnight before further analysis.

### Contact profilometry analysis of polymer arrays

A Dektak 6M stylus profilometer was used to determine gelation thickness response of all array spots. The stylus force was set to 1 mg with a scan speed of 120 μm/second. After aligning the stylus with each array row, a scan was initiated and an average height of each polymer array spot was determined and recorded.

### Cy3 fluorescence calibration of array slides

To enable fluorescence calibration, streptavidin-Cy3 conjugates were labeled on microarray slides identically to streptavidin-initiator conjugates as described earlier. Briefly, pre-printed bio-BSA microarray slides were first blocked with PBSA for 40 minutes, and then contacted with 25 μg/ml SA-Cy3 for 30 minutes. After rinsing with deionized water, fluorescence of Cy3 tagged microarrays were analyzed with an Affymetrix 428 array scanner. Scanner measurements were obtained at 30 db gain with 532 nm laser excitation and PMT detection with a bandpass filter centered at 570 nm. To determine the density of bound fluorophores, a Cy3 calibration slide (Full Moon Biosystems) was scanned at identical settings. The calibration slide consisted of 28 concentrations at a two-fold dilution, each with 12 replicates spots. A calibration curve was then generated relating fluorescence and fluorophore molecule surface density that was used to quantify photoinitiator binding.

### Photopolymerization of A549 cells using streptavidin-EITC photoinitiation

A human lung cancer cell line A549 was expanded in RPMI-1640 with 10% FBS and 1% penicillin/streptomycin. Cells were cultured to ~80% confluency and detached from the culture flask using trypsin/EDTA. 1.5 x 10^6^ cells in 1 mL were taken for polymerization. Cells were first washed twice in cold buffer (PBS 1X containing 3% FBS) by centrifuging at 500g for 3 mins at 4°C. The final cell pellet was resuspended in 200 μL of buffer having 2 μL of biotin mouse anti-human EGFR by gentle vortexing and incubated on ice for 40 mins. After 40 mins of incubation, cells were washed twice by centrifuging at 500g for 3 mins at 4°C in cold buffer. The cell pellet was then resuspended in 1 mL of cold buffer containing 25 μg/mL of streptavidin-eosin isothiocyanate (SA-EITC) and incubated for 30 mins on ice. The cells were then washed twice in cold PBS by centrifuging at 500*g* for 3 mins at 4°C. Monomer solution was prepared as described previously [16]. For the live cell studies, PEG diacrylate-3500 (M_n_ = 3,500 Da) is used in place of the shorter PEG diacrylate-575 (M_n_ = 575 Da) for improved cytocompatibility. Briefly, 25 wt% PEG diacrylate-3500, 21 mM triethanol amine, and 35 mM 1-vinyl-2-pyrrolidione were mixed thoroughly in PBS. The pH of the monomer solution was measured and adjusted to 7.5 using 1.2 M HCl. The monomer solution was bubbled with humidified ultra-pure nitrogen for 10 mins to remove dissolved oxygen and maintained at 4°C until usage. The cell pellet was resuspended in 300 μL of monomer solution containing 0.05 wt% of nile red, 20 nm fluorescent nanoparticles. The monomer cell solution was then loaded into a Chip-Clip well (Whatman) with a standard microscope slide (VWR) which was pre-cooled to 4°C. The Chip Clip was then placed in a sealed clear plastic bag. The bag with the Chip Clip was purged for an additional 10 mins. While still purging, photopolymerization reaction was initiated by turning on the LED lamp emitting 530 nm light at 30 mW/cm^2^ for 10 mins. After 10 mins, the cell suspension was collected into a 15 mL centrifuge tube and washed twice with PBS by centrifuging at 500g for 3 mins at 4°C.

### Photopolymerization of A549 cells using FITC-antibody photoinitiation

A549 cells were treated similarly as mentioned earlier for streptavidin-EITC photoinitiation. Cells were expanded to ~80% confluence and detached with trypsin. Approximately 1.5 x 10^6^ cells in 1mL were used for polymerization. Cells were first washed twice in cold buffer (1X PBS containing 3% FBS) by centrifuging at 500g for 3 mins at 4°C. The final cell pellet was resuspended in 200 μL of buffer and 2 μL of mouse anti-human EGFR by gentle vortexing and incubated on ice for 40 mins. After 40 mins of incubation, cells were washed twice by centrifuging at 500g for 3 mins at 4°C in cold buffer. The cell pellet was resuspended in 200 μL of buffer having 2 μL of FITC-horse anti-mouse IgG and incubated for another 40 minutes on ice. The cells were then washed twice in cold 1X PBS by centrifuging at 500g for 3 mins at 4°C. Photopolymerization steps were carried out in the same way as mentioned for the eosin Y photoinitiator.

### Cell viability–MTT assay

Cell viability after polymerization and lysis was examined with the MTT assay. Cells were initially suspended in media at a density of ~20,000 cells per 200 μL and added into each well of a 96 wells plate. 20 μL of Thiazolyl Blue Tetrazolium Bromide dissolved in PBS at a concentration of 5 mg/mL was added to the sample in the 96 wells plate and incubated for 3 h at 37°C. At the end of the incubation time period, the plate was centrifuged at 1000g for 5 mins. The supernatant was removed carefully and the sample was resuspended in 200 μL of DMSO. The sample was mixed well and the absorbance was measured at 570 nm.

### Flow cytometry

Cell fluorescence was measured using an Accuri C6 flow cytometer. Each sample was set to count for 10,000 events per run. A549 cells before and after FITC incubation was compared on FL1 channel (488 nm laser excitation with 530/30 BP filter detection) of the flow cytometer.

## Results and discussion

### Analysis of absorbance spectral overlap of xanthene photoinitiators

**Fig 1:**
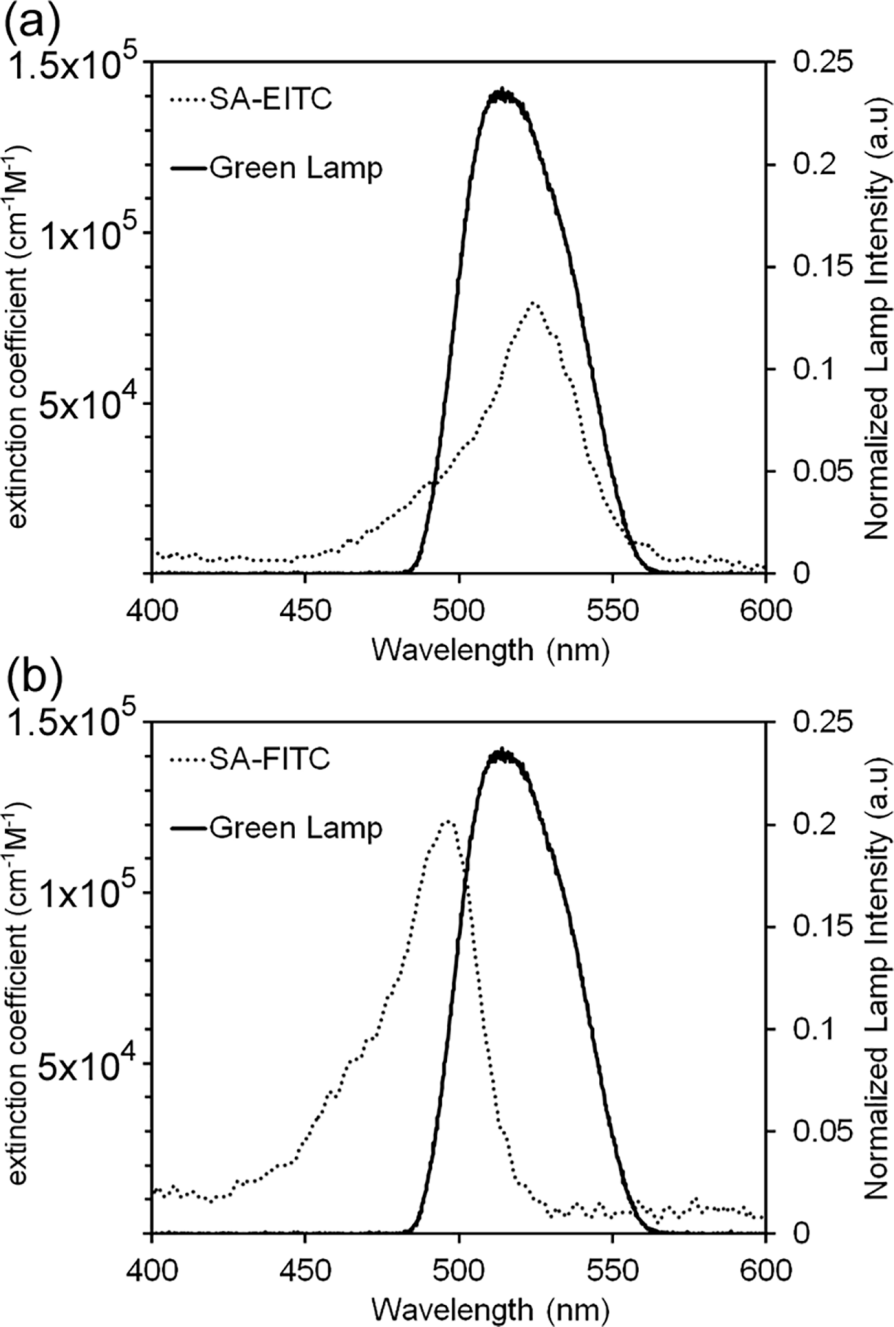
Analysis of photoinitiator absorbance spectral overlap. Overlap of (a) SA-FITC and (b) SA-EITC with a Thorlabs collimated LED emitting green light. Lamp spectra obtained from manufacturer.

For experimental consistency throughout this study, the same lamp source emitting visible spectrum light (maximum emission at approximately 530 nm) was used for all polymerization experiments. Several dyes in the xanthene family are well known to absorb visible-spectrum light leading to excitation to a triplet-state, which undergoes hydrogen abstraction with a tertiary amine. However, both the absorbance spectra and the photochemical efficiency of this transfer process is dependent on the structure and substitution within the xanthene molecule that differentiates the members of this class of dyes [5]. Eosin Y has a maximum excitation at approximately 530 nm, while fluorescein has a shifted maximum at approximately 495 nm (Fig 1). Though we previously have shown that the green LED lamp used in throughout this study is effective for eosin-mediated photopolymerization [16], the current study first sought to quantify the overlap and spectral mismatch for both eosin and fluorescein conjugates with the lamp used. The photoinitiator extinction coefficient dependency and normalized emitted light spectra are plotted versus light wavelength for both conjugates. Extinction coefficients values were calculated from absorbance values measured by UV-Vis spectroscopy using the Beer-Lambert law. Both streptavidin-eosin isothiocyanate (SA-EITC) and streptavidin-fluorescein isothiocyanate (SA-FITC) displayed extinction coefficient values consistent with that of free fluorescein and eosin Y dyes reported in the literature [27], suggesting that covalent protein labeling through an isothiocyanate linkage has a minimal effect on the light absorption of these molecules. By numerical integration, the overlap of each photoinitiator conjugate with the lamp emission was also calculated, which indicates the relative quantity of light absorbed for conjugate at identical concentrations. These calculations indicated that SA-EITC absorbs approximately 48% more light energy than SA-FITC with the 530 nm LED system. This trend was expected as the maxima of the LED lamp emission and SA-EITC absorbance are well aligned, while the SA-FITC has a larger lamp/photoinitiator mismatch. In addition to wavelength specific absorptivity, the quantum yield for triplet excitation, the rate of initiation, termination, and regeneration of the eosin will all play a role in the overall polymerization rate and coating gelation [5, 8, 25]. Here, we demonstrate the relative light absorbance presents an advantage to a polymerization initiation system using eosin over fluorescein for this lamp system.

### Microarray polymerization studies

**Fig 2:**
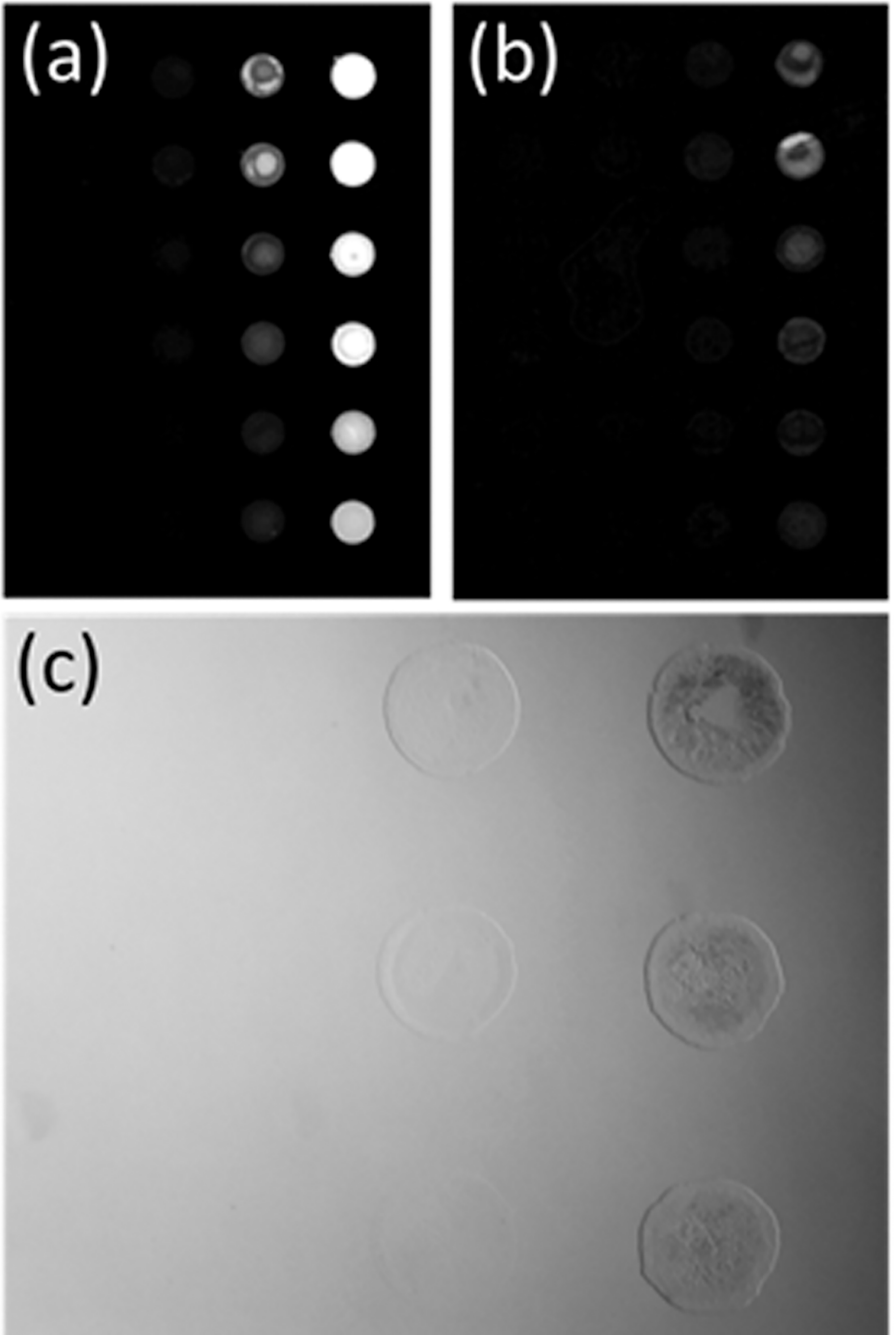
Specific polymerization of photoinitiator tagged microarrays. (A) Grayscale fluorescence scanner image of SA-EITC bound to biotin-BSA printed microarrays. Scan at 50 db gain with 532 nm excitation and 551 nm PMT detection with a 25 nm bandpass filter. (B) Grayscale fluorescence scanner image of SA-FITC bound to biotin-BSA printed microarrays. Scan at 50 db gain with 532 nm excitation and 551 nm PMT detection with a 25 nm bandpass filter. (C) Bright field optical microscopy example image of a microarray sample tagged with SA-FITC after photopolymerization at 30 mW/cm^2^ for 10 minutes.

The overall goal of this work was to quantitatively compare the potency of the xanthene dyes eosin and fluorescein to enact surface mediated photopolymerization at cell compatible conditions. We first use protein microarrays to monitor gelation response from an initiator tagged surface (Fig 2). Glass array slides are printed with serial dilutions of biotinylated-BSA and tagged with streptavidin-xanthene photoinitiators (SA-FITC or SA-EITC) that closely mimics initiator-tagged cell surfaces. Because of the high affinity between biotin-streptavidin [28], the density of photoinitiator is easily varied through dilution of the biotin content in biotin microarrays. The specific binding of SA-EITC and SA-FITC to biotin microarrays is shown in the fluorescence scanner images in panels 2a and 2b, respectively, where the fluorescent signal intensity of both photoinitiator conjugates is dependent on the serial dilution of printed biotin-BSA. This variation in fluorescent signal intensity indicates a systematic variation in the photoinitiator loading throughout the microarray. Fig 2C shows a representative bright field image of a microarray after photopolymerization at conditions that are shown to preserve high viabilities of cultured cells, above ~70%. The xanthene polymerization scheme of PEG diacrylate used here has been widely employed for rare protein detection in microassay formats and in conjunction with viable cells. In contrast to previously published monomer formulations for photopolymerized detection assays [12, 14, 21], this formulation has been slightly modified to support cell viability. Significant parameters affecting cell viability include physiological tonicity, pH, concentration of the triethanolamine coinitiator, and irradiation time. For cell compatible coating formulations, the monomer was an isotonic solution, phosphate buffered to pH 7.6, and the triethanol amine was reduced to 21 mM.

**Fig 3:**
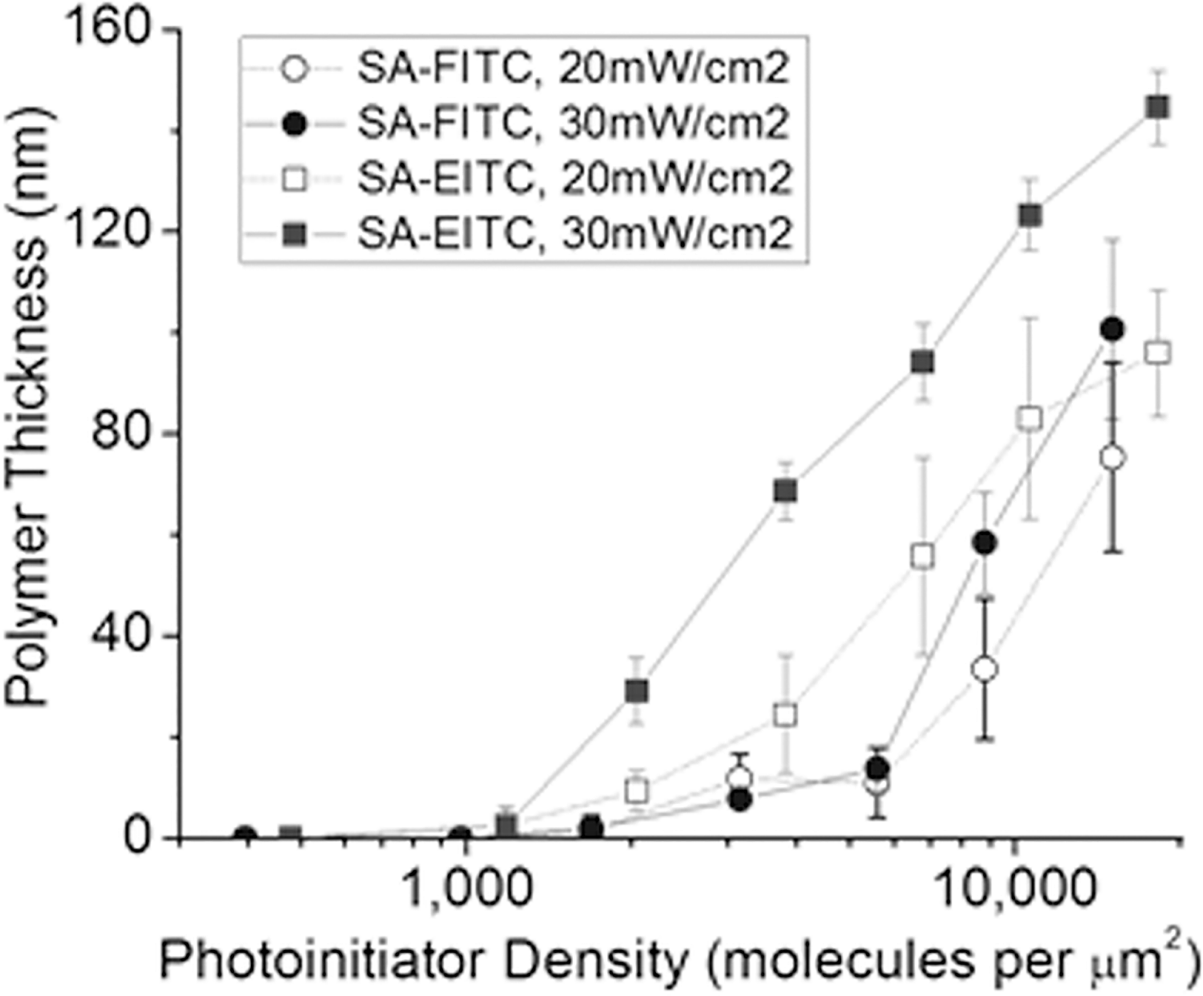
Comparative analysis of polymer gelation thickness vs. photoinitiator density for SA-FITC and SA-EITC tagged microarray samples. Analysis was conducted at cell-compatible formulation and reaction conditions consisting of 420 mM PEG-diacrylate, 21 mM triethanol amine, 35 mM vinyl pyrrolidone, in phosphate buffered media (pH = 7.5) with a constant reaction time of 10 minutes.

The thickness of the polymer coating resulting from varying surface densities of SA-FITC and SA-EITC at cell compatible conditions is provided in Fig 3. The effect of irradiation intensity and photoinitiator density on gelation thickness is also shown. Polymerizations initiated by 30 mW/cm^2^ and SA-EITC yielded a mean gelation thickness of 145 nm at the highest initiator labeling density studied of approximately 18,000 eosin molecules per μm^2^, which is consistent thickness with previous studies at similar polymerization conditions and photoinitiator densities [17, 26]. For SA-EITC, polymerization with the higher irradiation intensity of 30 mW/cm^2^ generally resulted in thicker films than irradiation with 20 mW/cm^2^, and this is most distinct above 2,000 molecules per μm^2^. In SA-FITC, there is minimal difference in film thickness with irradiation intensity. There is no observable dependence on light intensity for the minimum initiator density for measurable polymerization in either the SA-EITC or SA-FITC systems. For SA-EITC, this lower detection threshold is approximately 1,200 molecules per μm^2^, while SA-FITC is approximately 1,800 molecules per μm^2^.

SA-FITC generally yielded lower polymer thicknesses than SA-EITC at matched conditions, which was expected given the difference in relative light energy absorption for SA-EITC over SA-FITC (Fig 1). SA-FITC was still capable of generating appreciable polymer thicknesses, even though our relative absorption calculations indicate that SA-EITC absorbs 48% more photons than SA-FITC from the LED lamp source used throughout the study. At the higher labeling densities examined in the study of ~16,000 molecules per μm^2^, irradiation intensities of 30 mW/cm^2^ with SA-FITC initiation resulted in a mean gelation thickness of 100 nm. Previously reported photosensitization via fluorescein of similar aqueous polymerization systems indicated that fluorescein has significantly lower photoefficiencies that eosin both in radicalization from triplet quenching and in overall rate of polymerization [5]. These studies have largely been performed using high intensity, narrow bandwidth lasers well above 500 nm or with LEDs centered near 400 nm [25], which introduce considerable mismatch with the absorbance spectrum of fluorescein. Our results suggest that surface polymerization of PEG diacrylate coatings via SA-FITC labeling would further benefit from using lamp sources that are more highly aligned with the visible-range absorbance spectrum of FITC-protein conjugates.

**Fig 4:**
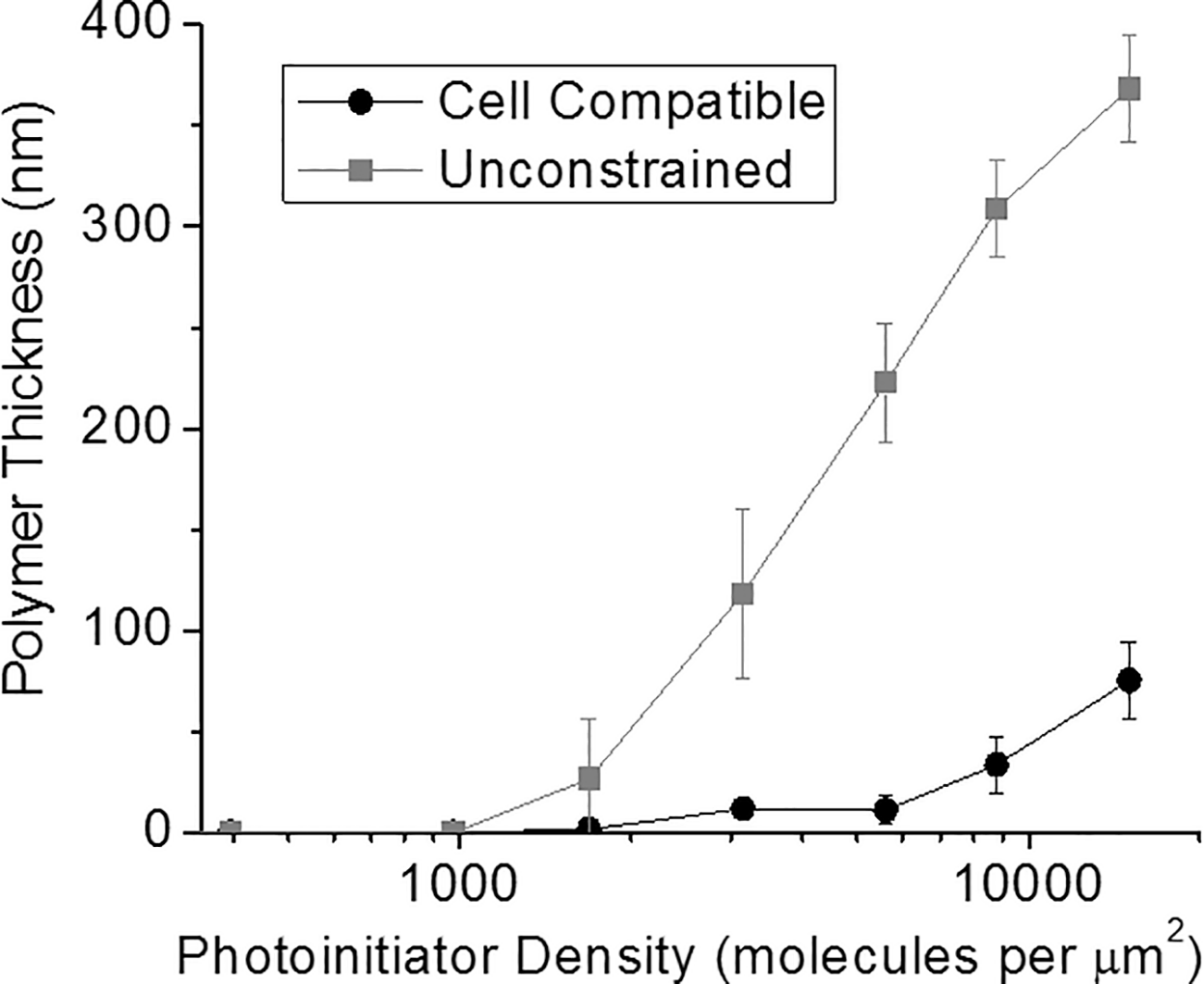
Comparison of polymer gelation thickness vs. photoinitiator density with cell compatible and unconstrained formulations and FITC photoinitiation. All samples were irradiated with 20mW/cm^2^ of green light (530 nm LED lamp, ThorLabs) for 10 minutes. Cell compatible = 420 mM PEG-diacrylate, 21 mM triethanol amine, 35 mM vinyl pyrrolidone, in phosphate buffered media (pH = 7.5). Unconstrained = 420 mM PEG-diacrylate, 210 mM triethanol amine, 35 mM vinyl pyrrolidone in deionized water.

We then investigated the impact of viable cell processing conditions on the FITC mediated polymerization. These “unconstrained” conditions were optimized for greatest hydrogel thickness by adjusting the coinitiator concentration to 210 mM TEA and the solution was not buffered or pH adjusted. Fig 4 shows a matched comparison of polymerization thickness for the unconstrained and cell compatible formulations. Polymerization thicknesses were drastically increased for the unconstrained formulation, and this increase in the amount of polymer formed is attributed to the coinitiator concentration being ten-fold higher than for the cell compatible formulation. Most notably, these FITC-initiated polymer responses rival those seen for previous reports of EITC initiation at similar labeling density and formulation scenarios. Further, the polymerization response increased consistently above the detection threshold at approximately 1,000 fluorescein molecules per μm^2^, which is desirable for a biodetection amplification system. These results support prior observations of fluorescein conjugates offering an alternative to the majority of current polymer-based amplification platforms that have been based on eosin labeling.

### Cell compatibility and feasibility of FITC-antibody labeling for surface coatings

**Fig 5:**
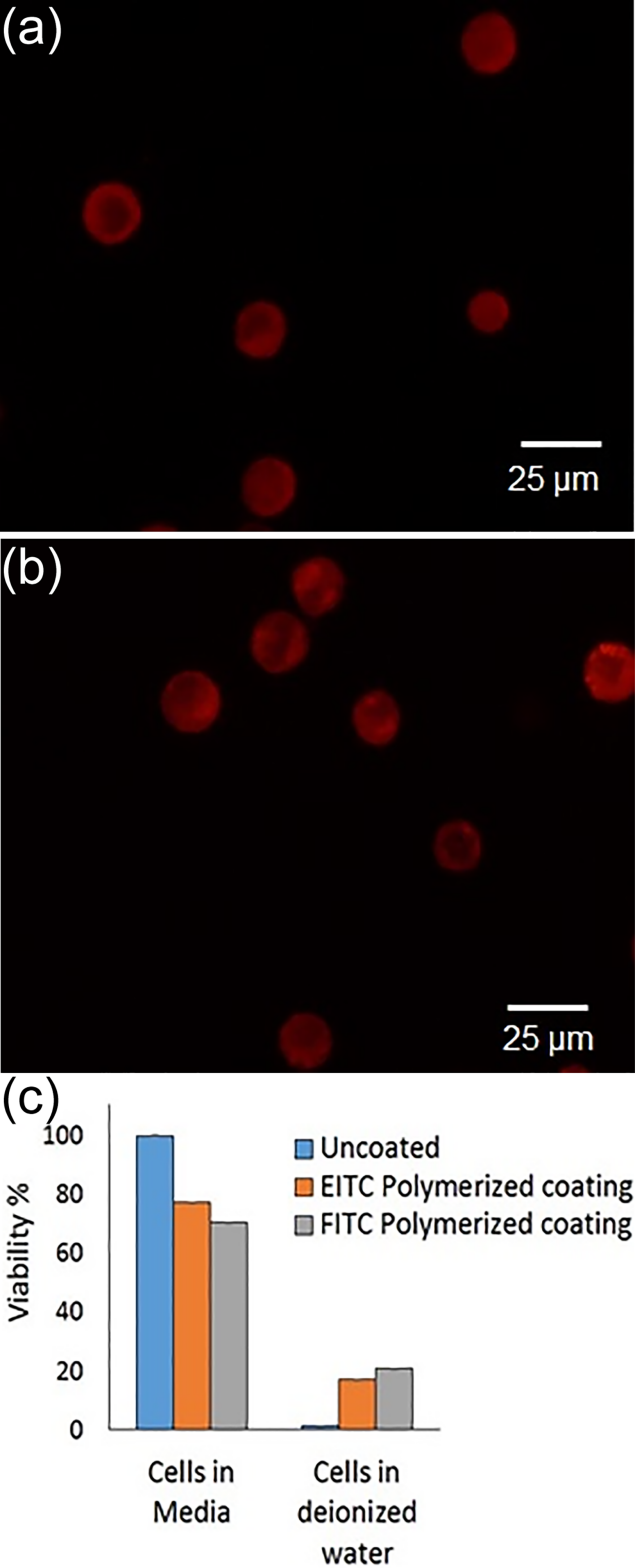
Polymerization of A549 cells using an eosin or fluorescein photoinitiator targeted to EGFR and PEGDA3500 as a monomer. (a) Fluorescence image of A549 cells coated with red fluorescent hydrogel using an eosin photoinitiator. (b) Fluorescence image of A549 cells coated with red fluorescent hydrogel using a flurescein photoinitiator. (c) Viability of A549 cells determined using MTT assay at various stages of polymerization (n = 4).

To confirm the cell compatibility of the polymerization conditions used in this study, we utilize the specific tagging the Epidermal Growth Factor Rector (EGFR, HER-1) on a non-small cell lung cancer cell line A549. This lung cancer line has been shown to highly express EGFR,[29] and represents a prototypic cell/receptor system for both the viability and feasibility study. In parallel, A549 populations specifically labeled with anti-EGFR were labeled with either SA-EITC or FITC-labeled secondary. SA-EITC labeling was confirmed by a 0.5-fold increase in flow cytometric mean FL1 signal, while FITC-secondary labeling exhibited a 12-fold increase in mean FL1 signal. After polymerization of PEG diacrylate in buffered, pH adjusted media, viability was assessed with a MTT assay, as shown in Fig 5. For both SA-EITC and FITC-secondary labeling, viability was maintained at approximately 70% after polymerization. While different cell lines and types can vary in their susceptibility to toxicity and each cell line should be individually assessed, these data support that the polymerization conditions used in the study are generally cell compatible and maintain high viability and cellular integrity. To confirm the presence of a conformal coating, samples were also exposed to conditions which lyse uncoated cells. PEG diacrylate coatings stabilize cells in lysing conditions, such as hypotonicity or surfactant exposure [16, 26]. Here, we hypotonically challenge our coated cells with excess deionized water. As previously observed, SA-EITC initiated coatings protected cells from osmotic lysis in deionized water while uncoated cells were lysed. The FITC-secondary initiated coatings exhibited a similar protection of the A549 cells from osmotic lysis, giving a comparable 20% viability of cells following a 10 minute deionized water exposure.

These results support that commercial FITC-labeled antibodies are capable of generating cell coatings at comparable viability as SA-EITC tagged cells. This protection correlates well with the microarray data of minimum density for polymerization being roughly comparable between the FITC and EITC systems, and highlights a disconnect between protection and overall polymer thickness. In all, this study supports the broad use of FITC-based probes in polymerization based amplification microassays and cell protective coatings for cell sorting,[16, 30] or nanoscale coatings for immunoisolation applications [7, 16, 17, 26].

## Conclusions

In this work, we compared the surface polymerization capabilities of two xanthene photoinitiator conjugates–SA-EITC and SA-FITC–in cell compatible environments. Our primary goal was to evaluate the feasibility of FITC cell tagging in the encapsulation of living mammalian cells as an alternative to SA-EITC tagging. Absorbance calculations indicated that FITC conjugates absorbed only 68% as much of the light dosage as EITC at matched irradiation conditions with a 530 nm LED lamp. Surprisingly, SA-FITC tagged microarrays were still capable of generating appreciable PEG diacrylate polymer thicknesses up to 100 nm, despite previous reports of fluorescein exhibiting drastically lower photoefficiencies as a type-II radical polymerization sensitizer [5]. An MTT viability assay indicated that both photoinitiator conjugates yielded high cell viabilities after cell surface polymerizations above 70%. As a preliminary feasibility experiment, polymerizing non-small cell lung cancer cells targeted with commercial FITC-labeled EGFR antibodies resulted in polymer formation that stabilized cells in hypotonic lysing conditions. Cytometry analysis indicated that 20% of cells were stabilized in lysing solutions, whereas our previous SA-EITC tagging strategy resulted in 19%. These results are encouraging as to the potential of FITC-antibodies for cell coatings.

## Supporting information

S1 Fig Plot of concentration vs. UV-vis spectral absorbance at 280 nm of streptavidin prepared in PBS 1X used to prepare standard curve by linear regression. (TIF)

S2 Fig Plot of concentration vs. UV-vis spectral absorbance at 530 nm of eosin-isothiocyanate prepared in PBS 1X used to prepare standard curve by linear regression. (TIF)

S3 Fig Plot of concentration vs. UV-Vis spectral absorbance at 495 nm of fluorescein-isothiocyanate prepared in PBS 1X used to prepare standard curve by linear regression. (TIF)

S4 Fig Plot of concentration vs. UV-Vis spectral absorbance at 550 nm of Cy3 prepared in PBS 1X used to prepare standard curve by linear regression. (TIF)

S5 Fig Calibration of Cy3 fluor per μm^2^ vs. scanner signal intensity for Cy3 microarray calibration slide (Full Moon BioSystems). (TIF)

S1 File Individual data points in an.xlsx file for Figs 3 and 4. (XLSX)

S2 File Individual data points in an.xlsx format for Fig 5. (XLSX)

S3 File Individual data points in an.xlsx format for S1 Fig, S2 Fig, S3 Fig, S4 Fig, and S5 Fig. (XLSX)
