# Supplementary figures and images for: Comparison of eosin and fluorescein conjugates for the photoinitiation of cell-compatible polymer coatings
Source: PLoS One. 2018 Jan 8;13(1):e0190880. doi: 10.1371/journal.pone.0190880 (PMC5757926; doi:10.1371/journal.pone.0190880)

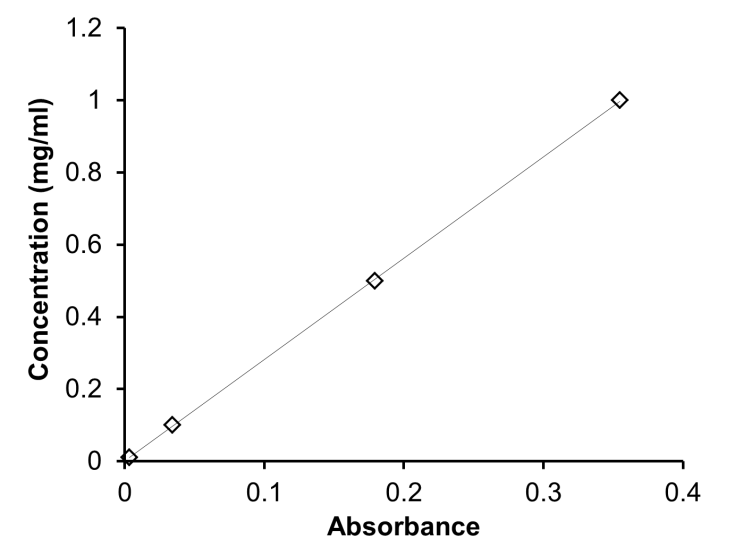

Supplement: S1 Fig — (TIF) [file pone.0190880.s001.tif]

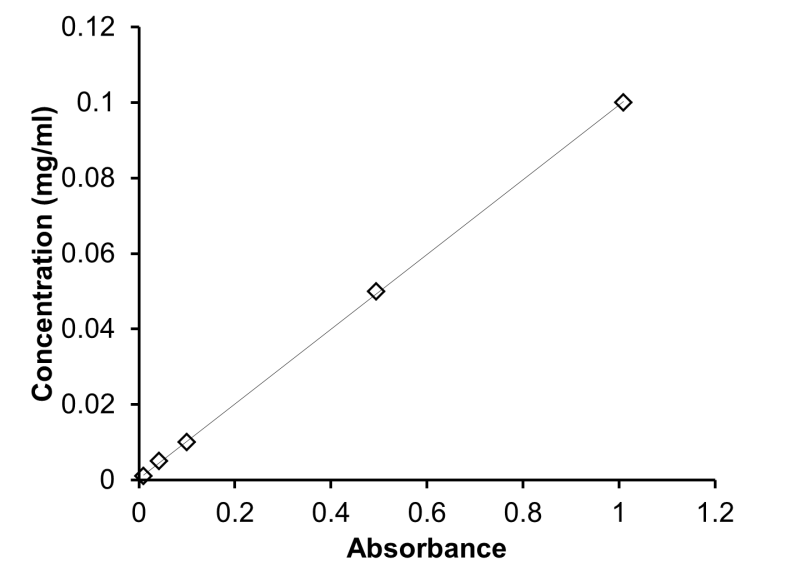

Supplement: S2 Fig — (TIF) [file pone.0190880.s002.tif]

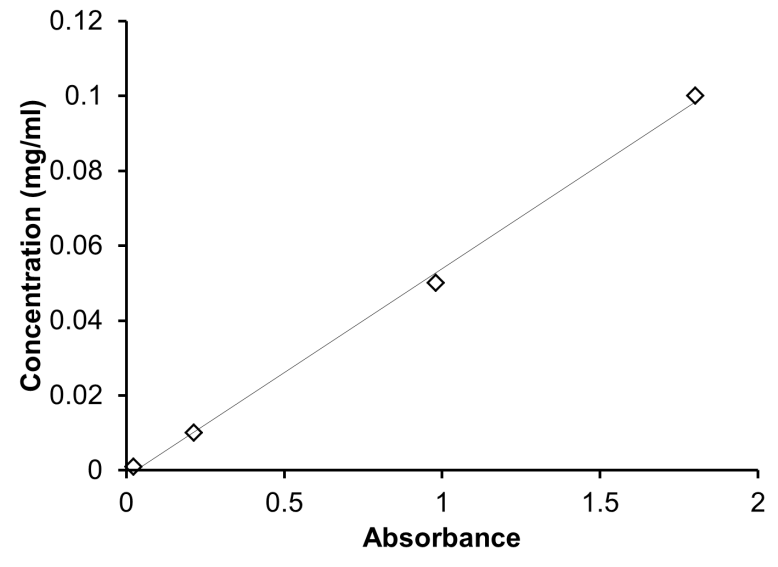

Supplement: S3 Fig — (TIF) [file pone.0190880.s003.tif]

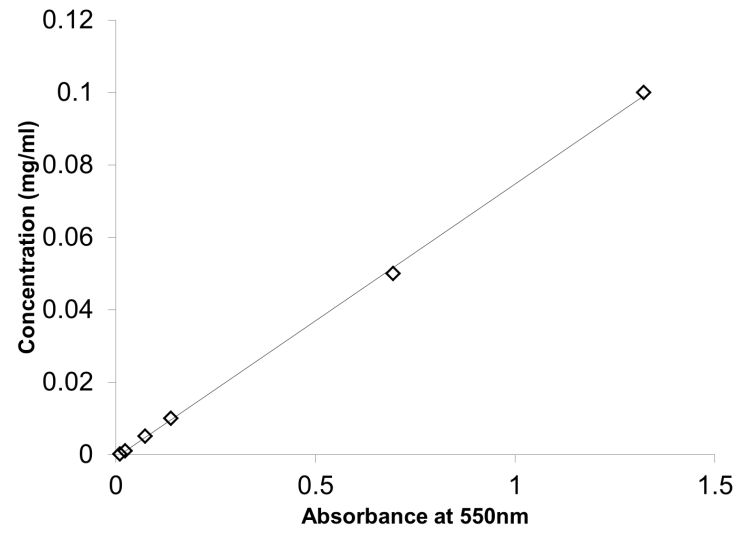

Supplement: S4 Fig — (TIF) [file pone.0190880.s004.tif]

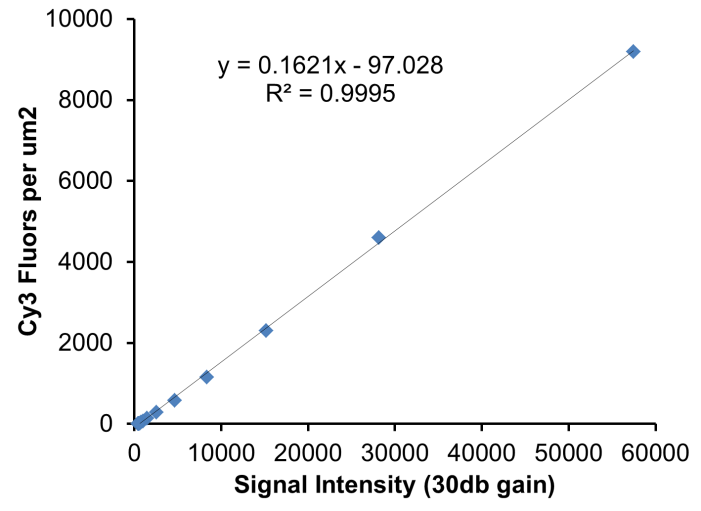

Supplement: S5 Fig — (TIF) [file pone.0190880.s005.tif]
